# Lymph node abscess caused by *Francisella tularensis* – a rare differential diagnosis for cervical lymph node swelling: a case report

**DOI:** 10.1186/s13256-019-2165-x

**Published:** 2019-08-09

**Authors:** R. Rothweiler, M. A. Fuessinger, R. Schmelzeisen, M. C. Metzger

**Affiliations:** Department of Oral and Maxillofacial Surgery Faculty of Medicine, University of Freiburg, Hugstetter Straße 55, 79106 Freiburg, Germany

**Keywords:** *Francisella tularensis*, Unilateral lymph node, Abscess, Infection

## Abstract

**Background:**

Cervical lymph node swelling is quite a common symptom mainly caused by infections in the face or as metastasis of a malignant tumor. In infection cases in particular, rare diseases should never be overlooked. With an incidence of 120 cases in the United States of America (USA) and approximately 25 cases in Germany per year, infection with the pathogen *Francisella tularensis* is one of these rare diseases that can cause massive lymph node swellings and might even be fatal.

**Case presentation:**

The example of a healthy 67-year-old German woman who was treated at our university hospital presents a typical progression of a localized form of tularemia. The pathogen could be identified in a universal 16S ribosomal deoxyribonucleic acid (DNA) polymerase chain reaction. Pathogen-specific treatment with lymph node abscess incision, daily rinsing of the abscess cavity, and orally administered antibiotic therapy with doxycycline could cure our patient completely without any remaining complications.

**Conclusion:**

In patients with cervical lymph node swelling caused by infection it is indispensable to perform specific identification of the pathogen for further local and specific antibiotic treatment. Possible infections with atypical bacteria like *Francisella tularensis* should never be ignored. Universal polymerase chain reactions are a suitable method for early detection of such rare pathogens.

## Background

Cervical lymph node swelling is found in many different disciplines from general medicine to specialized disciplines such as otorhinolaryngology or craniomaxillofacial surgery. Reasons for the swelling might be benign or malign diseases. Benign swellings or even abscesses as a result of infections are mostly caused by *Staphylococcus aureus* and group A streptococcus. A rare zoonotic disease also causing lymph node swelling is tularemia. This disease appears all over the northern hemisphere but the incidence of only 1056 cases recorded in the European Union in 2016 is quite low [1]. *Francisella tularensis*, the pathogen of tularemia, is a Gram-negative bacterium; it was first described in 1911 in the United States of America (USA). The bacterium can be divided into four different subspecies. The subspecies *F*. *tularensis* subspecies *holarctica* is mostly prevalent in Europe whereas the subspecies *F*. *tularensis* subspecies *tularensis* is most frequently found in North America [2]. Although the bacterium itself could be identified in more than 250 different animal species, the exact transmission path to humans is not quite clear yet. One possible path of infection beside infection through wild animals seems to be transmission through mosquitos [3]. Another possible way could be infection through fruit, such as the recently described transmission path of freshly pressed grape must [4]. Two different forms of clinical appearance must be distinguished: a localized (outer) and a systemic (inner) form. The most obvious form in Europe is the outer form here represented by the so-called ulceroglandular disease. After an incubation time of 3 to 5 days nonspecific systemic symptoms such as fever, chills, anorexia, and malaise appear; the portal of entry presents as a single ulcerative lesion with a central eschar. Sometimes this lesion is not visible due to location in the hair. At the same time or a little later, regional lymph node swelling or even lymph node abscesses occur. Known complications without adequate treatment are persisting lymph node swelling with fever attacks at night, meningitis, or even organ damage, for example of the kidneys or the heart. Depending on the subspecies of infection, mortality rates vary a lot; whereas infections with *F*. *tularensis* subspecies *holarctica* show a low mortality rate, this rate increases up to 30% in infections caused by *F*. *tularensis* subspecies *tularensis* if no sufficient treatment ensues [5]. In order to avoid serious illness and complications, early adequate treatment after identification of the pathogen is necessary. Effective antibiotic substances are aminoglycosides, fluoroquinolones, tetracycline, chloramphenicol, and rifampicin. Erythromycin as representative of the macrolides should not be used due to the natural resistance especially of the *F*. *tularensis* subspecies *holarctica* [6]. Drainage is an additional and urgently needed treatment especially when abscesses are obvious.

## Presentation of case

**Fig. 1:**
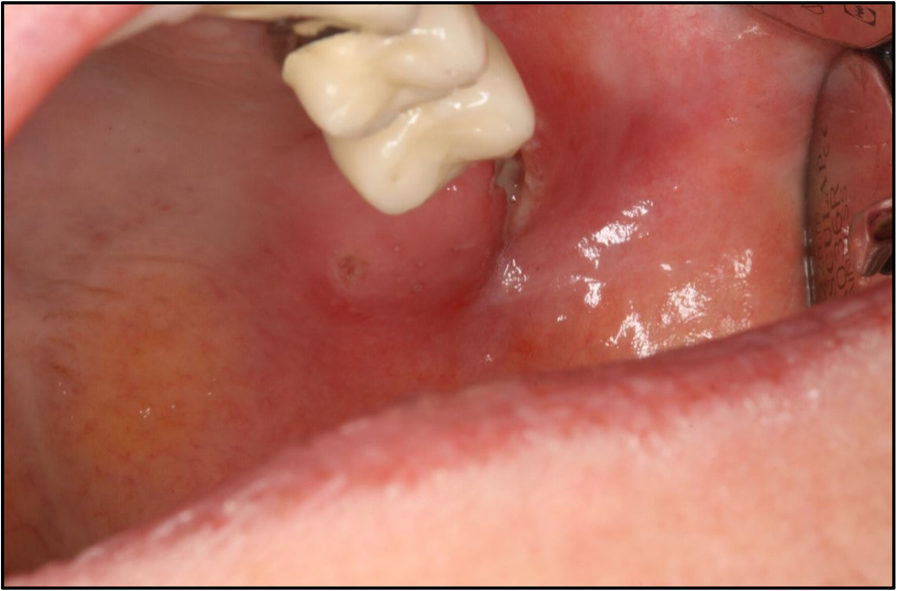
Image of the local laceration in the upper left wisdom tooth region

**Fig. 2:**
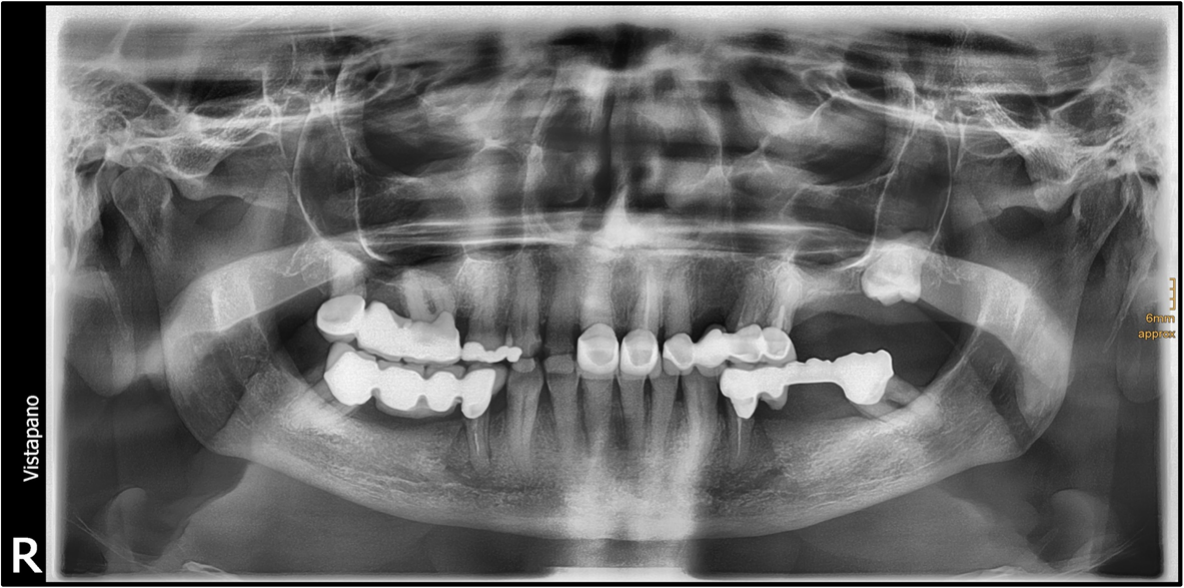
Panoramic radiograph before tooth extraction

A 67-year-old German woman without any pre-existing illnesses was assigned to us by colleagues of the Ear, Nose, and Throat (ENT) department with an intraoral laceration in the region of her upper left wisdom tooth. She felt faint and had quite high fever. Her leukocytes were not increased (5.88 × 10^3^/μl), whereas a massive elevation of C-reactive protein (224 mg/l) could be detected. Therapy consisting of intravenously administered antibiotic treatment with penicillin V 10 mega units once daily was started. In addition to that the impacted tooth was extracted the same day (Figs. 1 and 2).

**Fig. 3:**
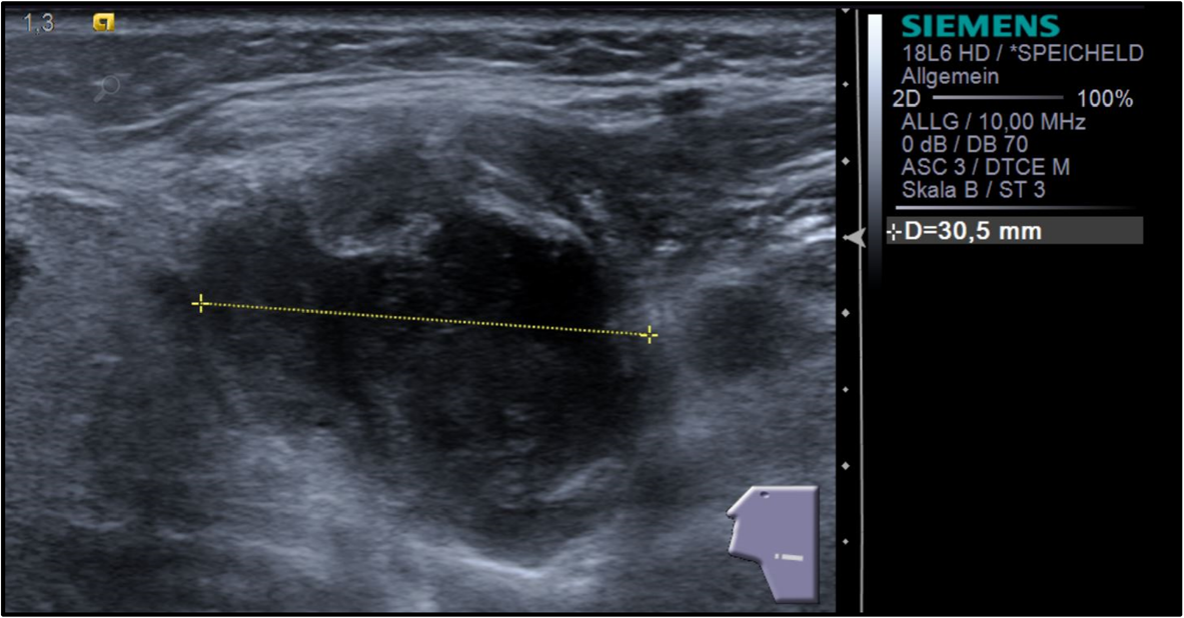
Sonographical image showing the extremely increased lymph nodes 25 days after tooth resection

This treatment regimen did not significantly improve her health status. She started to get higher fever accompanied by an increase in cervical lymph node swelling. At 25 days after tooth removal, the size of her lymph nodes increased to a diameter of approximately 30 mm. She was hospitalized for a repeated intravenous treatment with antibiotics consisting of penicillin V 10 mega units once daily combined with metronidazole 0.5 g twice daily again (Fig. 3).

**Fig. 4:**
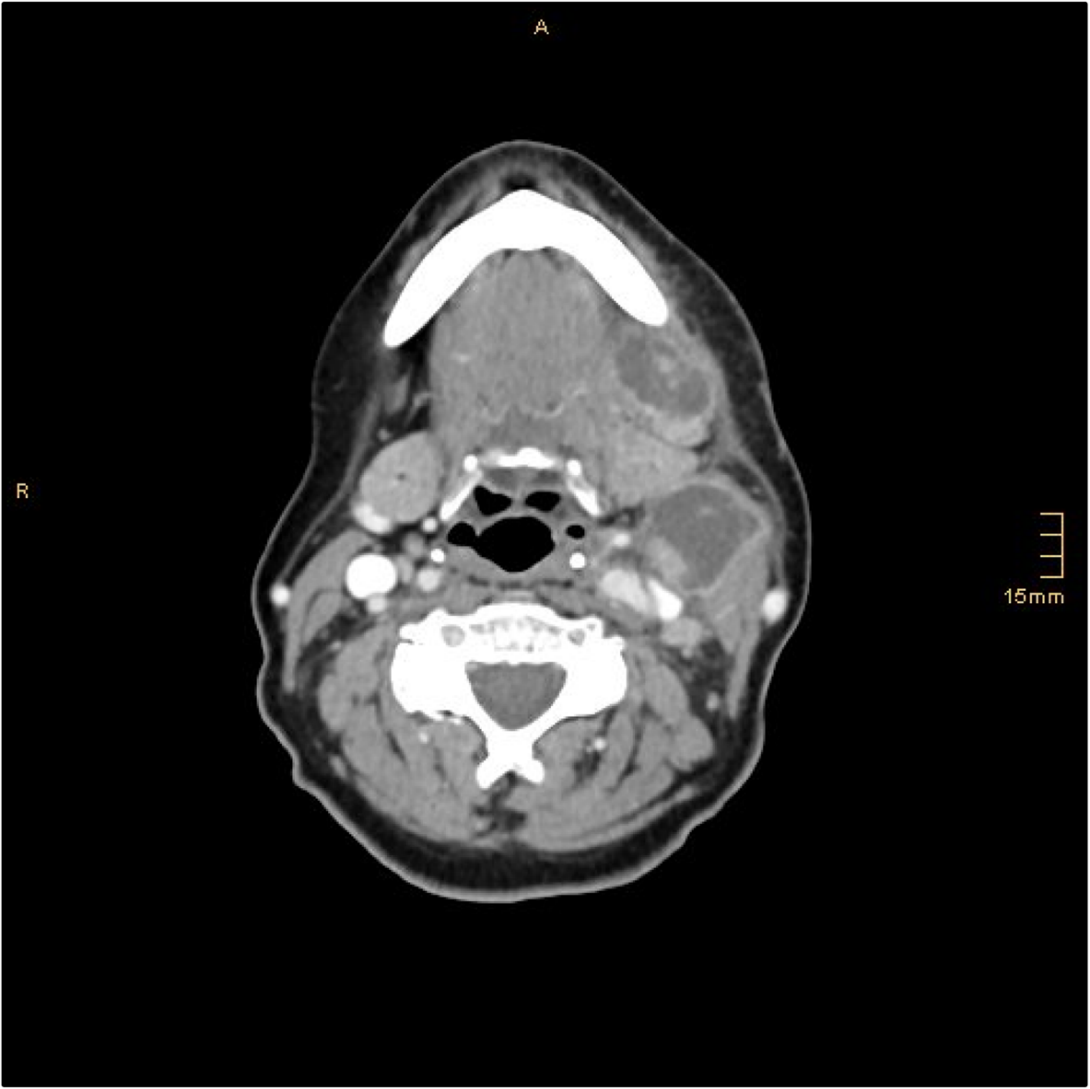
Computed tomography image of the abscess formations in the left submandibular region

During this in-patient treatment our patient’s health condition continued to worsen. Computed tomography (CT) imaging with contrast agent was done to have a more precise look at her lymph nodes and the pathologies she presented in her neck area. In this CT imaging, two big “meltings” could be detected in the area of the former lymph nodes (Fig. 4).

As abscesses were now visible, an incision and drainage were done under general anesthesia via an extraoral approach. A sample for a microbiological investigation was taken and tubes for further abscess drainage were inserted. In the sample taken, the pathogen *F. tularensis* subspecies *holarctica* could be identified 5 days later by polymerase chain reaction (PCR). In a universal 16S ribosomal deoxyribonucleic acid (rDNA) setup the pathogen could be detected. Subsequent tests with specific quantitative PCR (qPCR) for FopA and Tul4 confirmed the results. The antibiotic regime was changed in consultation with colleagues from the Department of Infectiology to an oral therapy with doxycycline 100 mg twice a day for 14 days.

**Fig. 5:**
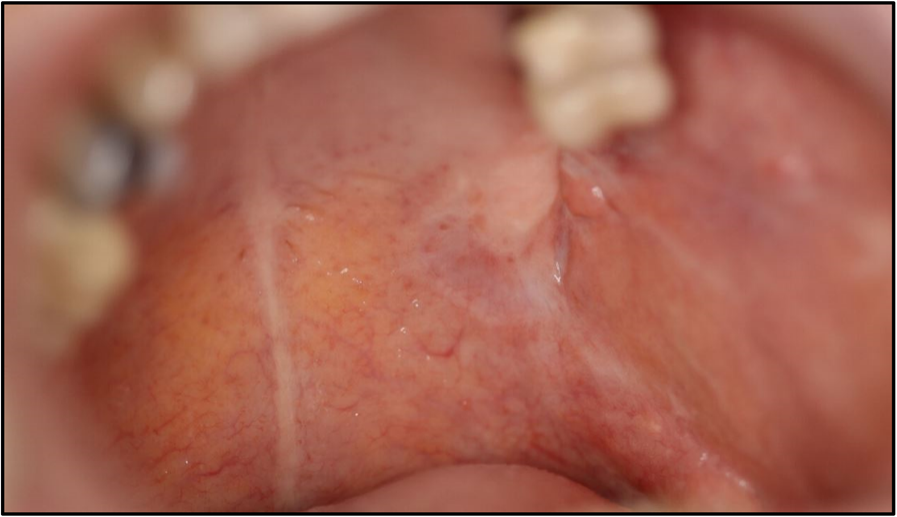
Irritation-free scar in the former left upper wisdom tooth region half a year after the infection

Under this regime and daily rinsing of the abscess caverns, our patient recovered fast. At day 33 after tooth removal she could be discharged in a good condition with laboratory parameters in a normal range (leukocytes 8.54 × 10^3^/μl; C-reactive protein 16 mg/l). In a follow-up consultation 4 weeks later at our Department of Infectiology, the complete recovery of our patient could be certified. No further treatment was needed. During a further appointment for control half a year later, she reported no new appearance of symptoms. On an intraoral examination, an irritation-free scar was still visible (Fig. 5, Table 1).

**Table 1:** Important milestones in progression of disease

Day	Treatment
0	First contact with patient; wisdom tooth extraction and intravenously administered antibiotic administration consisting of penicillin V 10 mega units once daily
25	Restart of a treatment with intravenously administered antibiotic administration consisting of penicillin V 10 mega units once daily combined with metronidazole 0.5 g twice daily
28	Computed tomography (CT) imaging and abscess incision from an extraoral approach
32	Diagnosis of tularemia; change of antibiotic regime to doxycycline 100 mg twice a day orally
33	Discharged from hospital
46	Out-patient follow-up

## Discussion

Approximately 600 lymph nodes are located in the human body among which the submandibular, the axillary, or inguinal regions are palpable in healthy conditions [7]. Cervical lymphadenopathy as a pathological condition is a symptom that appears often in adults as well as in children, with a rate of up to 45% of children showing palpable lymphadenopathy [8]. Reasons for this swelling can vary from malignant to non-malignant causes. Whereas most lymph node swellings in children are caused by infections the rate of lymphadenopathies due to malignant reasons increases with age [9]. Mean odds are stated between 1.03 and 1.05 for each 10-year increase [9, 10]. The main bacterial pathogens of suppurative cervical adenitis are described to be infections with *S. aureus* and group A streptococcus [11, 12]. Other pathogens, such as typical or atypical mycobacteria, *Bartonella henselae*, or *F. tularensis* are more seldom but should never be ignored as differential diagnosis.

On clinical examination, lymph nodes in the neck area greater than 1 cm are described as enlarged, except for the jugulodigastric nodes; here, 1.5 cm is accepted as normal size [13, 14]. Signs of inflammation, pain when pressing on the nodes, and insufficient movability on the underground, are further pathological markers that are not seen under healthy conditions. In particular, insufficient movability is a predictive marker for rupture of the capsule of the node in malignant processes. Additional so-called malignant B-symptoms such as fever, weight loss, or night sweats are often present at the same time [15].

A first algorithm for structural evaluation of patients with lymphadenopathy was proposed in 1978 by Greenfield and Jordan [16]. Although these suggestions have been criticized a lot, basic elements are still used in examination workflows today. In a diagnostic procedure, detailed anamnesis should always be the first step. Practitioners often get first hints for the cause of swelling. Anamnesis should be followed by clinical examination. Special attention should be paid to visible extraoral or intraoral changes, such as scars (typical for *Bartonella* infections), decayed teeth, or malignant formations. More than half of the diagnoses can be made with these modalities [17]. Blood analysis (at least blood count, C-reactive protein, ± procalcitonin/interleukin-6) completes these initial steps.

Medical ultrasound of the neck still remains the method of choice for instrument-based examination, especially of superficial cervical lymph nodes. Ultrasound is widely available and has no ionizing radiation. It might be superior to other imaging methods in differentiating metastatic from non-metastatic nodes using Doppler sonography [18]. The disadvantages are a low penetration depth and big quality differences caused by experience of the investigators. For the detection of deep cervical nodes, such as those of the retropharyngeal space, a CT scan should always be done. It is first choice for a survey and follow-up of metastatic nodes in the neck [19]. It is also the method of choice for defining accurate localization of enlarged nodes and their relationship to surrounding structures. Magnetic resonance imaging (MRI) as a radiation-free method provides high soft-tissue contrast resolution for morphologic evaluation of lymph nodes and their relationships. Diffusion-weighted imaging enables benign lymph nodes to be distinguished from malignant lymph nodes [20]. Due to its limited availability and high price it should not be used as a standard diagnosis algorithm except for examination of children.

Histopathological evaluation of progress should follow as a diagnostic in persisting lymph node pathologies. Open excision biopsy of lymph nodes is considered to be the gold standard especially in the diagnosis of malignant lymphoma [21]. Methods such as fine-needle aspiration or cutting needle biopsy are less invasive, less time consuming, and can be done under local anesthesia. Due to advances in immunohistochemical and cytopathological methods their accuracy has increased in the last few years but never reached the accuracy of excision especially in enlarged lymph nodes showing a heterogeneous pattern of disease [22]. In metastasis diagnosis of lymph nodes or in examination of deep-seated lymph nodes with close proximity to vital structures, such as mature blood vessels and nerves, excision biopsy should remain the method of choice [23].

For the disease of tularemia all the diagnostic methods mentioned above are useful. Detailed anamnesis often reveals contact with animals in the past or patients to be farmers or hunters with close contact with wild animals. Scars in the face, mouth, or the hair might be visible as portals of entry in the ulceroglandular form in addition to a massive lymph node swelling in the neck region. Indirect diagnostic methods such as serum screenings are not precise especially in the first 2 weeks after primary infection. Early diagnosis, therefore, needs to be done by direct identification of the pathogen using molecular biological methods such as Enzyme-linked immunosorbent assay (ELISA) or different PCR methods like reverse transcriptase (RT)-PCR or even 16S rDNA PCR which shows a lower sensitivity compared to conventional RT-PCR [24]. Sample material for investigation might be achieved from swabs of the ulcerative lesion. Enlarged lymph nodes or “meltings” in lymph nodes should be illustrated by ultrasound examination. Additional CT imaging can be used for a more detailed localization of the suppurative lymph nodes or empyemas especially in advance of possible drainage. This intervention should always be combined with a direct reconfirming identification of the pathogen.

## Conclusion

Cervical lymph node swelling as a symptom appears quite often in old and young people. As reasons vary a lot and range from malignant to non-malignant causes, an adequate diagnostic procedure is needed. In a common diagnostic setting, anamnesis should be the first step and should be followed by non-invasive investigation methods such as ultrasound, CT, or MRI imaging methods. Long-lasting lymph node swellings or melted lymph node abscesses should additionally be examined with the use of histological or microbiological analysis methods in order to initialize a specific therapy.

Although the bacterial pathogens that cause lymph node enlargement are well known in the common clinical setting, an infection with uncommon pathogens should always be taken into consideration in order to start a specific therapy. One of these pathogens is infection with *F. tularensis*; *F. tularensis* is the bacteria that causes the disease tularemia and is commonly transmitted by wild animals. Without any treatment the disease reveals a mortality rate of up to 30% in infections caused by the *F*. *tularensis* subspecies *tularensis*. Severe complications can be avoided by an early and specific treatment consisting of a pathogen-specific antibiotic treatment combined with drainage of possible abscesses. Early identification of the pathogen is indispensable.
